# Identification of Source of *Brucella suis* Infection in Human by Whole-Genome Sequencing, United States and Tonga

**DOI:** 10.3201/eid2201.150843

**Published:** 2016-01

**Authors:** Christine Quance, Suelee Robbe-Austerman, Tod Stuber, Tom Brignole, Emilio E. DeBess, Laurel Boyd, Brad LeaMaster, Rebekah Tiller, Jenny Draper, Sharon Humphrey, Matthew M. Erdman

**Affiliations:** National Veterinary Services Laboratories, Ames, Iowa, USA (C. Quance, S. Robbe-Austerman, T. Stuber, M.M. Erdman);; United States Department of Agriculture Animal Plant Health Inspection Service, Tumwater, Washington, USA (T. Brignole);; Oregon Health Authority Public Health Division, Portland, Oregon, USA (E.E. DeBess, L. Boyd);; Oregon Department of Agriculture, Salem, Oregon, USA (B. LeaMaster);; Centers for Disease Control and Prevention, Atlanta, Georgia, USA (R. Tiller);; Animal Health Laboratory, Ministry for Primary Industries, Wallaceville, New Zealand (J. Draper, S. Humphrey)

**Keywords:** *Brucella suis*, brucellosis, whole-genome sequencing, genomic epidemiology, zoonoses, boar, One Health, Tonga, Polynesia, Oregon, United States

## Abstract

*Brucella suis* infection was diagnosed in a man from Tonga, Polynesia, who had butchered swine in Oregon, USA. Although the US commercial swine herd is designated brucellosis-free, exposure history suggested infection from commercial pigs. We used whole-genome sequencing to determine that the man was infected in Tonga, averting a field investigation.

In August 2013, a man in his 20s from Tonga, Polynesia, who had moved to the United States in June 2010, was examined in a hospital in Portland, Oregon after experiencing 4 weeks of fever, night sweats, headache, productive cough, shortness of breath, and weight loss. He also reported pleuritic chest pain and abdominal pain radiating to his back. A computed tomography scan showed lung and liver abnormalities. Blood cultures grew *Brucella suis* biovar 1. After treatment with oral sulfamethoxazole (800 mg 3×/d), trimethoprim (160 mg 3×/d), and doxycycline (100 mg 1×/d); and intravenous gentamicin (580 mg 3×/d), the infection resolved. Because *Brucella* infection is a reportable condition in Oregon, the case was referred to the Oregon Health Authority Acute and Communicable Disease office, and authority personnel informed the Oregon Department of Agriculture’s veterinary officials that the patient had routinely purchased pigs from a local farm for home slaughter, suggesting the patient may have contracted *B. suis* from commercial swine.

The US commercial swine herd is considered to be free of *B. suis*; however, *B. suis* is endemic among feral swine and occasionally has infected domestic swine (*1*,*2*). Slaughter surveillance, primarily by using the buffered acidified plate antigen test, is conducted routinely to identify such events to prevent the re-establishment of *B. suis* in the commercial swine herd and to protect workers. 

## The Study

Serum from sows culled on the farm in question had been collected during routine slaughter surveillance. Two weakly positive results during the previous 3 years were investigated by following the guidelines in the USDA Swine Brucellosis Control/Eradication State-Federal-Industry Uniform Methods and Rules (*3*); no brucellosis was confirmed. Although feral swine reside in Oregon, none had recently been reported near the farm. 

The case-patient’s lack of exposure to feral swine and a known exposure to commercial swine required further investigation. An epidemiologic investigation to evaluate the commercial herd’s infection status would require testing of swine on the premises and related farms, and tasks such as tracing sales from the herd and testing swine possibly exposed to swine brucellosis by temporary movement of boars to or from farms for breeding purposes. Such investigations can be costly, especially if there has been extensive movement of swine in and out of the herd.

Whole-genome sequencing (WGS) and single-nucleotide polymorphism (SNP) analyses can provide increased resolution to identify the source of infections without conducting more expensive field investigations (*4*). The National Veterinary Services Laboratories (NVSL) had implemented WGS and SNP analysis as the primary means of genotyping *B. abortus* and *Mycobacterium bovis* isolates and applied this information to identify sources of other outbreaks. Although a project to sequence *B. suis* isolates from animals of US origin was in process at the time of this investigation, few had been sequenced. To rapidly investigate the case in Oregon, laboratory staff from the Centers for Disease Control and Prevention extracted and provided DNA from the isolate recovered from the Oregon patient, and the NVSL sequenced 59 *B. suis* biovar 1 isolates recovered from US-origin animals. Oregon was declared free of swine brucellosis in 1987 (http://www.oregon.gov/ODA/programs/AnimalHealthFeedsLivestockID/AnimalDiseases/Pages/AnimalDiseases.aspx), and the NVSL did not have archived isolates from Oregon or surrounding states. Consequently, isolates selected for sequencing were mostly from the southern United States and had been recovered from domestic swine and cattle during 1993–2013 (Technical Appendix 1). A few isolates were selected from dogs, horses, and humans, all of whom, based on data from epidemiologic investigations, likely had contact with feral swine.

To obtain the whole-genome sequences, we sequenced *B. suis* DNA on a MiSeq instrument (Illumina, San Diego, CA, USA) using 2×250 paired-end chemistry and the Nextera XT library preparation kit (Illumina), targeting 100% coverage. FASTQ files from the instrument were put through the NVSL in-house analysis pipeline (https://github.com/USDA-VS). Reads were aligned to *B. suis* isolate 1330 (GenBank Reference Sequence accession nos. NC_017250 and NC_017251) by using BWA (*5*) and Samtools (*6*). We processed BAM files (*6*) by using the Genome Analysis Toolkit best-practice workflow (*7*). SNPs were called by using the UnifiedGenotyper from the toolkit, outputting SNP to variant call files (*7*–*9*). Results were filtered by using a minimum Phred (http://www.1000genomes.org/node/101) quality score (scaled probability of SNP presence) of 300 and allele count of 2. From the variant call files, SNPs gathered were output in 3 formats: an aligned FASTA file (http://www.ncbi.nlm.nih.gov/BLAST/blastcgihelp.shtml); a tab-delimited file with the position location and SNPs grouped and sorted; and a phylogenetic tree created by using RAxML (*10*). We visually validated SNPs using the Integrative Genomics Viewer (*11*). Sequencing files were deposited in the National Center for Biotechnology Information Sequence Read Archive (SRA) under the Bioproject PRJNA251693 http://www.ncbi.nlm.nih.gov/bioproject/?term=PRJNA251693 (online Technical Appendix 1). 

Initially, as evidenced by the 60 isolates described, the United States could not be ruled out as a source because of a lack of resolution. Two approaches were considered to improve the resolution of the *B. suis* database: sequence enough isolates originating in the United States to assess the likelihood that any US-origin isolate would closely match, or sequence isolates originating from Tonga and determine whether they were clustered with the isolate from the case-patient in Oregon. The difficulty in obtaining isolates from representative feral swine throughout the United States precluded the first option as a viable solution. To obtain isolates from Tonga, we contacted the Ministry of Primary Industries in New Zealand for assistance; its staff members provided DNA from 7 *B. suis* isolates recovered from patients who were from Tonga. New Zealand is not known to have *B. suis* in its feral or commercial swine populations; therefore, humans with diagnoses of *B. suis* had likely been infected in another country. 

We constructed a maximum-likelihood phylogenetic tree comprising the Oregon human isolate, the 59 field isolates from the United States, and the 7 isolates from New Zealand recovered from patients from Tonga (Figure). The branch labeled as OutGroup_suis3 roots the phylogenetic tree, and the A node is the most recent common ancestor (MRCA) for all isolates. Three lineages evolved from the MRCA. Initially, the Oregon human isolate was the only representative in its lineage. Without the perspective of the Tonga isolates, a US source for this isolate could not be ruled out because it shared the same MRCA as 2 other lineages occurring within the United States. All additional Tonga–origin isolates clustered tightly with the Oregon human isolate share a common ancestor at the B node. The Oregon human isolate is anchored by 2 additional common ancestors: C and D. In addition to the phylogenetic tree, a table displaying divergent SNPs of closely related isolates, including nucleotide calls and the positions within the genome, was created for transparency and clarity (Technical Appendix 2). Often, 1 or 2 SNP calls inform the epidemiology of a case. For example, the 08-924 isolate recovered from a patient in Tonga in 2008 has 1 additional SNP (a thymine at position 1809039 on chromosome 2) from sharing a common ancestor with the Oregon isolate.

**Figure:**
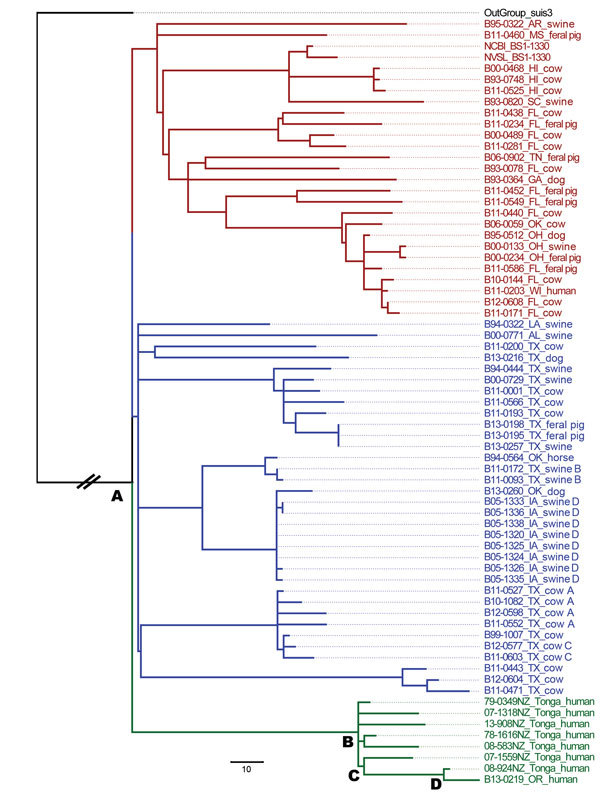
Maximum-likelihood phylogenetic tree of *Brucella suis* isolates from the United States and Tonga. The phylogenetic tree was rooted using a truncated *B. suis* biovar 3 isolate (black text). Red and blue text indicate 59 isolates recovered from US origin sources. Green text indicates the isolate recovered from the immigrant from Tonga residing in Oregon, B13-0219, and 7 additional isolates recovered from patients from Tonga in New Zealand. The first 2 digits of the sample number indicate the year isolated. Isolates recovered from different animals within a herd are labeled with the same letter designation after the species information. The letter A designates the common ancestor between all isolates; B, C, and D identify the common ancestors between the Tonga and Oregon isolates. Scale bar indicates 10 single-nucleotide polymorphisms.

## Conclusions

WGS and SNP analysis effectively concluded that this case-patient was infected in Tonga and not by swine in the United States. Thus, widespread testing of domestic swine was not conducted; agricultural trade continued without restrictions, and postexposure treatment of contacts participating in home slaughter or meat preparation was not needed. This case also demonstrates the value of and need for an international database of validated WGS isolates that can be used by both human and animal health officials in their respective and collaborative epidemiologic investigations. Finally, this case highlights the benefits of a One Health (http://onehealthinitiative.com/) approach between public and animal health, including state, federal, and international authorities.

**Technical Appendix 1.** Identification of the source of *Brucella* *suis* infection of a human by comparison of host characteristics and genetic sequences in samples from the human case-patient to those of animals and humans in the United States and Tonga.

**Technical Appendix 2.** Divergent single-nucleotide polymorphism of closely related isolates from persons the United States and Tonga.

## References

[1] Wyckoff AC, Henke SE, Campbell TA, Hewitt DG, VerCauteren KC. Feral swine contact with domestic swine: a serologic survey and assessment of potential for disease transmission. J Wildl Dis. 2009;45:422–9. 10.7589/0090-3558-45.2.422 19395751

[2] United States Department of Agriculture Animal and Plant Health Inspection Service. Swine brucellosis status map. APHIS. 2011 Jul [cited 2015 Jan 11]; http://www.aphis.usda.gov/wps/wcm/connect/294e846c-3d5f-4031-b5a6-500c283ad650/br_status_map_257.jpg?MOD=AJPERES

[3] United States Department of Agriculture Animal and Plant Health Inspection Service. Swine brucellosis control/eradication: state–federal–industry uniform methods and rules. APHIS. 1998 Apr [cited 2015 Sep 13]. https://www.aphis.usda.gov/animal_health/animal_dis_spec/swine/downloads/sbruumr.pdf

[4] Kao RR, Haydon DT, Lycett SJ, Murcia PR. Supersize me: how whole-genome sequencing and big data are transforming epidemiology. Trends Microbiol. 2014;22:282–91. 10.1016/j.tim.2014.02.011 24661923 PMC7125769

[5] Li H, Durbin R. Fast and accurate short read alignment with Burrows-Wheeler transform. Bioinformatics. 2009;25:1754–60. 10.1093/bioinformatics/btp324 19451168 PMC2705234

[6] Li H, Handsaker B, Wysoker A, Fennell T, Ruan J, Homer N, The sequence alignment/map format and SAMtools. Bioinformatics. 2009;25:2078–9. 10.1093/bioinformatics/btp352 19505943 PMC2723002

[7] Van der Auwera GA, Carneiro MO, Hartl C, Poplin R, del Angel G, Levy-Moonshine A, From FASTQ data to high confidence variant calls: the Genome Analysis Toolkit best practices pipeline. Curr Protoc Bioinformatics. 2013 Oct 15; 11(1110):11.10.1–11.10.33 10.1002/0471250953.bi1110s43 PMC4243306 25431634

[8] DePristo MA, Banks E, Poplin R, Garimella KV, Maguire JR, Hartl C, A framework for variation discovery and genotyping using next-generation DNA sequencing data. Nat Genet. 2011;43:491–8. 10.1038/ng.806 21478889 PMC3083463

[9] McKenna A, Hanna M, Banks E, Sivachenko A, Cibulskis K, Kernytsky A, The Genome Analysis Toolkit: a MapReduce framework for analyzing next-generation DNA sequencing data. Genome Res. 2010;20:1297–303. 10.1101/gr.107524.110 20644199 PMC2928508

[10] Stamatakis A. RAxML version 8: a tool for phylogenetic analysis and post-analysis of large phylogenies. Bioinformatics. 2014;30:1312–3 and. 10.1093/bioinformatics/btu033 24451623 PMC3998144

[11] Robinson JT, Thorvaldsdottir H, Winckler W, Guttman M, Lander ES, Getz G, Integrative genomics viewer. Nat Biotechnol. 2011;29:24–6. 10.1038/nbt.1754 21221095 PMC3346182

