# Supplementary material for: Identification of Source of Brucella suis Infection in Human by Whole-Genome Sequencing, United States and Tonga
Source: Emerg Infect Dis. 2016 Jan;22(1):79–82. doi: 10.3201/eid2201.150843 (PMC4696693; doi:10.3201/eid2201.150843)
Supplement: Supplementary file 1 — Technical Appendix 1. Identification of the source of Brucella suis infection of a human by comparison of host characteristics and genetic sequences in samples from the human case-patient to those of animals and humans in the United States and Tonga. [file 15-0843-Techapp-s1.pdf]

Article DOI: http://dx.doi.org/10.3201/eid2201.150843

Identification of the Source of *Brucella suis* Infection of a Human by Using Whole Genome Sequencing, United States and Tonga

Technical Appendix 1

Technical Appendix 1 Table. Characteristics of hosts and *Brucella suis* isolates from the United States and Tonga

Isolate identification/origin	Host country of origin	Host country of residence	Host state of residence	Host county or parish of residence	Host species	Year recovered	Bioproject accession no.	Biosample accession no.	SRA run	Mean coverage	Genome coverage (%)
NVSL_BS1_1330	N/D	N/D	N/D	N/D	N/D	N/D	PRJNA251693	SAMN03768240	SRR2058923	163.663X	100
B93-0078_FL_Cow	USA	USA	FL	Osceola	Cattle	1993	PRJNA251693	SAMN03768289	SRR2058972	178.572X	100
B93-0364_GA_Dog	USA	USA	GA	Ben Hill	Dog	1993	PRJNA251693	SAMN03768290	SRR2058973	114.465X	100
B93-0748_HI_Cow	USA	USA	HI	Hawaii	Cattle	1993	PRJNA251693	SAMN03768291	SRR2058974	133.207X	100
B93-0820_SC_Swine	USA	USA	SC	Richland	Pig	1993	PRJNA251693	SAMN03768292	SRR2058975	148.28X	100
B94-0322_LA_Swine	USA	USA	LA	Washington	Pig	1994	PRJNA251693	SAMN03768293	SRR2058976	158.633X	100
B94-0444_TX_Swine	USA	USA	TX	Robertson	Pig	1994	PRJNA251693	SAMN03768294	SRR2058977	167.435X	100
B94-0564_OK_Horse	USA	USA	OK	Atoka	Horse	1994	PRJNA251693	SAMN03768295	SRR2058978	42.2018X	99.99
B95-0322_AR_Swine	USA	USA	AR	Pope	Pig	1995	PRJNA251693	SAMN03768296	SRR2058979	164.585X	100
B95-0512_OH_Dog	USA	USA	OH	Lawrence	Dog	1995	PRJNA251693	SAMN03768297	SRR2058980	159.536X	100
B99-1007_TX_Cow	USA	USA	TX	Kleberg	Cattle	1999	PRJNA251693	SAMN03768298	SRR2058981	114.116X	100
B00-0133_OH_Swine	USA	USA	OH	Washington	Pig	2000	PRJNA251693	SAMN03768241	SRR2058924	144.365X	100
B00-0234_OH_Feralpig	USA	USA	OH	Washington	Pig, feral	2000	PRJNA251693	SAMN03768242	SRR2058925	156.42X	100
B00-0468_HI_Cow	USA	USA	HI	Hawaii	Cattle	2000	PRJNA251693	SAMN03768243	SRR2058926	157.994X	100
B00-0489_FL_Cow	USA	USA	FL	Okeechobee	Cattle	2000	PRJNA251693	SAMN03768244	SRR2058927	32.8519X	99.99
B00-0729_TX_Swine	USA	USA	TX	Navarro	Pig	2000	PRJNA251693	SAMN03768245	SRR2058928	110.543X	100
B00-0771_AL_Swine	USA	USA	AL	Clarke	Pig	2000	PRJNA251693	SAMN03768246	SRR2058929	97.1904X	100
B05-1320_IA_SwineD	USA	USA	IA	Louisa	Swine	2005	PRJNA251693	SAMN03768247	SRR2058930	165.883X	100
B05-1324_IA_SwineD	USA	USA	IA	Louisa	Swine	2005	PRJNA251693	SAMN03768248	SRR2058931	134.984X	100
B05-1325_IA_SwineD	USA	USA	IA	Louisa	Swine	2005	PRJNA251693	SAMN03768249	SRR2058932	144.038X	100
B05-1326_IA_SwineD	USA	USA	IA	Louisa	Swine	2005	PRJNA251693	SAMN03768250	SRR2058933	144.209X	100
B05-1333_IA_SwineD	USA	USA	IA	Louisa	Swine	2005	PRJNA251693	SAMN03768251	SRR2058934	216.366X	100
B05-1335_IA_SwineD	USA	USA	IA	Louisa	Swine	2005	PRJNA251693	SAMN03768252	SRR2058935	146.144X	99.87
B05-1336_IA_SwineD	USA	USA	IA	Louisa	Swine	2005	PRJNA251693	SAMN03768253	SRR2058936	177.157X	100
B05-1338_IA_SwineD	USA	USA	IA	Louisa	Swine	2005	PRJNA251693	SAMN03768254	SRR2058937	109.356X	100
B06-0059_OK_Cow	USA	USA	OK	Blaine	Cattle	2005	PRJNA251693	SAMN03768255	SRR2058938	87.2441X	100
B06-0902_TN_Feralpig	USA	USA	TN	UNK-NL	Pig, Feral	2006	PRJNA251693	SAMN03768256	SRR2058939	157.207X	99.93
B10-0144_FL_Cow	USA	USA	FL	Hardee	Cattle, Holstein	2009	PRJNA251693	SAMN03768257	SRR2058940	193.056X	100
B10-1082_TX_CowA	USA	USA	TX	Kleberg	Cattle	2010	PRJNA251693	SAMN03768258	SRR2058941	219.891X	100
B11-0001_TX_Cow	USA	USA	TX	Henderson	Cattle	2010	PRJNA251693	SAMN03768259	SRR2058942	144.575X	100

Isolate identification/origin	Host country of origin	Host country of residence	Host state of residence	Host county or parish of residence	Host species	Year recovered	Bioproject accession no.	Biosample accession no.	SRA run	Mean coverage	Genome coverage (%)
B11-0093_TX_SwineB	USA	USA	TX	Titus	Pig, Hampshire	2010	PRJNA251693	SAMN03768260	SRR2058943	172.609X	100
B11-0171_FL_Cow	USA	USA	FL	Hardee	Cattle, Holstein	2010	PRJNA251693	SAMN03768261	SRR2058944	211.483X	100
B11-0172_TX_SwineB	USA	USA	TX	Titus	Pig, Yorkshire	2010	PRJNA251693	SAMN03768262	SRR2058945	120.728X	100
B11-0193_TX_Cow	USA	USA	TX	Robertson	Cattle	2010	PRJNA251693	SAMN03768263	SRR2058946	165.877X	100
B11-0200_TX_Cow	USA	USA	TX	Cherokee	Cattle	2011	PRJNA251693	SAMN03768264	SRR2058947	131.654X	100
B11-0203_WI_Human	Laos	USA	WI	N/D	Human	2010	PRJNA251693	SAMN03768265	SRR2058948	182.251X	100
B11-0234_FL_Feralpig	USA	USA	FL	Palm Beach	Pig, feral	2011	PRJNA251693	SAMN03768266	SRR2058949	190.261X	100
B11-0281_FL_Cow	USA	USA	FL	Okeechobee	Cattle, Holstein	2011	PRJNA251693	SAMN03768267	SRR2058950	287.636X	100
B11-0438_FL_Cow	USA	USA	FL	Pasco	Cattle	2011	PRJNA251693	SAMN03768268	SRR2058951	184.45X	99.99
B11-0440_FL_Cow	USA	USA	FL	Hardee	Cattle	2011	PRJNA251693	SAMN03768269	SRR2058952	199.548X	100
B11-0443_TX_Cow	USA	USA	TX	Liberty	Cattle	2011	PRJNA251693	SAMN03768270	SRR2058953	215.99X	100
B11-0452_FL_Feralpig	USA	USA	FL	Polk	Pig, feral	2011	PRJNA251693	SAMN03768271	SRR2058954	161.119X	100
B11-0460_MS_Feralpig	USA	USA	MS	Bolivar	Pig, feral	2011	PRJNA251693	SAMN03768272	SRR2058955	187.912X	100
B11-0471_TX_Cow	USA	USA	TX	Washington	Cattle	2011	PRJNA251693	SAMN03768273	SRR2058956	200.059X	100
B11-0525_HI_Cow	USA	USA	HI	Hawaii	Cattle, Angus	2011	PRJNA251693	SAMN03768274	SRR2058957	184.934X	100
B11-0527_TX_CowA	USA	USA	TX	Kleberg	Cattle	2011	PRJNA251693	SAMN03768275	SRR2058958	124.746X	100
B11-0549_FL_Feralpig	USA	USA	FL	Marion	Pig, feral	2011	PRJNA251693	SAMN03768276	SRR2058959	21.9847X	100
B11-0552_TX_CowA	USA	USA	TX	Kleberg	Cattle	2011	PRJNA251693	SAMN03768277	SRR2058960	175.004X	100
B11-0566_TX_Cow	USA	USA	TX	Mason	Cattle	2011	PRJNA251693	SAMN03768278	SRR2058961	43.4882X	99.99
B11-0586_FL_Feralpig	USA	USA	FL	Hillsborough	Pig, feral	2011	PRJNA251693	SAMN03768279	SRR2058962	196.554X	100
B11-0603_TX_CowC	USA	USA	TX	Hildago	Cattle	2011	PRJNA251693	SAMN03768280	SRR2058963	131.457X	100
B12-0577_TX_CowC	USA	USA	TX	Hidalgo	Cattle	2012	PRJNA251693	SAMN03768281	SRR2058964	309.082X	100
B12-0598_TX_CowA	USA	USA	TX	Kleberg	Cattle	2012	PRJNA251693	SAMN03768282	SRR2058965	145.342X	100
B12-0604_TX_Cow	USA	USA	TX	Washington	Cattle	2012	PRJNA251693	SAMN03768283	SRR2058966	158.646X	100
B12-0608_FL_Cow	USA	USA	FL	Hardee	Cattle, Holstein	2012	PRJNA251693	SAMN03768284	SRR2058967	172.491X	100
B13-0195_TX_Feralpig	USA	USA	TX	Houston	Pig, feral	2013	PRJNA251693	SAMN03768285	SRR2058968	181.158X	100
B13-0198_TX_Feralpig	USA	USA	TX	Houston	Pig, feral	2013	PRJNA251693	SAMN03768286	SRR2058969	197.406X	100
B13-0216_TX_Dog	USA	USA	TX	Wood	Dog, American bulldog	2013	PRJNA251693	SAMN03768287	SRR2058970	171.956X	100
B13-0219_OR_Human	Tonga	USA	OR	N/D	Human	2013	PRJNA251693	SAMN03768288	SRR2058971	207.254X	100
B13-0257_TX_Swine	USA	USA	TX	Houston	Pig	2013	PRJNA251693	SAMN03768299	SRR2058982	144.993X	100
B13-0260_OK_Dog	USA	USA	OK	Canadian	Dog	2013	PRJNA251693	SAMN03768300	SRR2058983	168.06X	100
79-0349NZ_Tonga_Human	Tonga	New Zealand	N/A	N/A	Human	1979	PRJNA251693	SAMN03768301	SRR2058984	192.373X	100
78-1616NZ_Tonga_Human	Tonga	New Zealand	N/A	N/A	Human	1978	PRJNA251693	SAMN03768302	SRR2058985	167.943X	100
07-1318NZ_Tonga_Human	Tonga	New Zealand	N/A	N/A	Human	2007	PRJNA251693	SAMN03768303	SRR2058986	177.514X	99.99
07-1559NZ_Tonga_Human	Tonga	New Zealand	N/A	N/A	Human	2007	PRJNA251693	SAMN03768304	SRR2058987	173.205X	100

Isolate identification/origin	Host country of origin	Host country of residence	Host state of residence	Host county or parish of residence	Host species	Year recovered	Bioproject accession no.	Biosample accession no.	SRA run	Mean coverage	Genome coverage (%)
08-583NZ_Tonga_Human	Tonga	New Zealand	N/A	N/A	Human	2008	PRJNA251693	SAMN03768305	SRR2058988	177.836X	100
08-924NZ_Tonga_Human	Tonga	New Zealand	N/A	N/A	Human	2008	PRJNA251693	SAMN03768306	SRR2058989	151.22X	100
13-908NZ_Tonga_Human	Tonga	New Zealand	N/A	N/A	Human	2013	PRJNA251693	SAMN03768307	SRR2058990	208.926X	100

N/D, no data available; N/A, not applicable.
Sequencing files were deposited in the National Center for Biotechnology Information Short Read Archive under the Bioproject PRJNA25169.
